# 

**Published:** 1841

**Authors:** 


### IMPORTANT ANNOUNCEMENT.

Having been informed by the Editors of this Journal, that John Trenor, M. D. of this city, has so far misinterpreted the Secretary's minutes of the proceedings of the American Society of Dental Surgeons, published in the last number of this work, as to understand that he is therein declared to have been *personally present* at the first meeting of said society;—this is to inform our numerous readers and all whom it may concern, agreeably with Doctor Trenor's request, that I am as happy to state, as he possibly can be to have it known, that he was not *personally* present on that occasion.

SOLYMAN BROWN,

*Recording Secretary, No.* 13 *Park Place, New-York.*

*January* 15*th*, 1841.

### NOTICE.

As several of the popular Essays in the form of tracts, ordered by the American Society of Dental Surgeons, are in the hands of the Secretary, having been forwarded by their authors, to be stereotyped and put to press, at the expense of the Society, this is to inform all the members, or others who may desire any of these essays for private distribution, that they may be had of the subscriber at the rate of two dollars per hundred previously to the meeting of the Society in August next, when a tariff of charges will probably be established. Cash, in all instances must accompany the order, and letters must be post paid by the writers. After August next, it is presumed that every member of the Society, who has paid his annual dues, will be entitled to a certain number of these tracts, in virtue of his membership; and that they will be sold to order, at a small advance above cost price.

SOLYMAN BROWN, Secretary,

*No.* 13. *Park Place, New-York.*

*January,* 1841.

### OF OUR NEXT VOLUME.

---

As the issue of one more double number will complete the first volume of this Journal, it becomes necessary to apprize our numerous patrons, in this country and Europe, that the second volume will appear in four quarterly numbers, of an average of 150 pages each number, at the subscription price of $5 per volume. It is intended to issue the entire volume in one year, inasmuch as contributions of important matter are crowding upon us from all quarters, and our subscribers in every direction encourage us to proceed in our novel undertaking, assuring us of their continued support.

In regard to the advance of subscription, from Three to Five Dollars, the publishers need only say that the present volume is decidedly the cheapest periodical ever published, and has depended for support mainly on the contributions of our special patrons, as noted on the third page of our cover, to which list we now add the name of Mr. I. I. Greenwood, of this city, who recently enclosed to the New York Editor, and through him to the Treasurer, the liberal sum of One Hundred Dollars, as a perpetual subscriber. The donations of our special patrons are confined to the first volume of our work, and therefore it becomes necessary to provide for its future support by raising the price of subscription from Three to Five Dollars.

Inasmuch as the issue of the first number of the second volume will be in some measure controlled by the condition of our subscription list, the Publishing Committee respectfully invite those gentlemen of the profession, who intend to aid in our undertaking, by becoming subscribers, to forward their names and the amount of subscription as early as convenient, in order that the first number may make its appearance early in the spring, so that the second number may contain the proceedings of the American Society of Dental Surgeons, at its second annual meeting, in August next;—at which time, it will be remembered, the members of that Association assemble, agreeably to adjournment, in Philadelphia.

### RECENT PUBLICATIONS.

The following works we had intended to notice in the present Number of the Journal, but the multiplicity of our engagements prevents us from doing it. They shall, however, receive early attention.

Researches on the Development, Structure, and Diseases of the Teeth, By Alexander Nasmyth, F. L. S. F. G. S., &c &c.—*London*, 1839.

A Practical Treatise on the Human Teeth, showing the cause of their destruction, and the means of their Preservation. By William Robertson, with Plates—*London*, 1839.

Popular Treatise on the Structure and Diseases of the Human Teeth, with an Illustration of the present state of Dental Mechanism. By J. L. Murphy—*London*, 1837.

Preservation of the Teeth, a Family Guide, being familiar observations on their Structure and Diseases, &c. &c. By David R. Hitchcock, Surgeon Dentist.—*Boston*, 1840.

A Treatise on the Anatomy and Physiology of the Teeth, &c. &c.—their Diseases and Treatment, with Practical Observations on Artificial Teeth, and Rules for their construction. By David Wemyss, M. R. C. S. E., &c. &c.—*London*, 1835.

### OUR SUBSCRIPTION.

The following is a synopsis of our present subscription list, showing the States to which our patrons belong. It will be seen by inspecting the table, that the State of New-York furnishes nearly one half of our patronage; and that from five of the States we have as yet had no subscribers.

As Mr. Koecker, of London, has consented that we should reprint, in our second volume, his celebrated work on Dental Surgery, the Publishing Committee anticipate a large augmentation of subscriptions even from those States which have come so promptly to our aid in this first volume of our work. And when it becomes generally known that we have the promise of assistance from the pens of some of our most talented brethren in England, as well as from others who have never yet contributed to our pages in this country, there will scarcely remain a doubt of the promptness of the profession, generally, in sending in their names at an early day as subscribers to the second volume, accompanied by the subscription price of Five Dollars, safely deposited in a post-paid letter, addressed to one of the Editors, either of the members of the Publishing Committee, or to the Treasurer, Mr. Jahial Parmly, No. 3 Bond Street, New-York.

As many of our present subscribers have not remitted the amount of their subscriptions, even at this eleventh hour, the Publishing Committee find themselves compelled to adhere strictly to their rule, and not forward any numbers of the second volume to any subscriber whose amount of subscription is not paid punctually in advance. It is vastly more convenient for each individual who wishes to take the Journal, to enclose five dollars in a letter, and drop it into the post-office, than for the Publishing Committee to pay the printer, and give a general credit to their subscribers.

### SUBSCRIPTION FROM THE SEVERAL STATES.

| | *No. of Subscribers.* | *No. of Copies.* |
|---|---|---|
| Maine, | 0 | 0 |
| New Hampshire, | 3 | 3 |
| Vermont, | 0 | 0 |
| Massachusetts, | 15 | 16 |
| Rhode Island, | 3 | 3 |
| Connecticut, | 15 | 15 |
| New-York, | 91 | 312 |
| New Jersey, | 6 | 6 |
| Pennsylvania, | 34 | 72 |
| Delaware, | 0 | 0 |
| Maryland, | 30 | 109 |
| Virginia, | 30 | 57 |
| North Carolina, | 13 | 16 |
| South Carolina, | 9 | 9 |
| Georgia. | 11 | 12 |
| Alabama, | 3 | 3 |
| Mississippi, | 0 | 0 |
| Louisiana, | 8 | 30 |
| Arkansas, | 2 | 5 |
| Tennessee, | 5 | 5 |
| Kentucky. | 16 | 17 |
| Missouri, | 2 | 3 |
| Indiana, | 2 | 2 |
| Illinois, | 2 | 2 |
| Michigan, | 0 | 0 |
| Ohio, | 11 | 11 |
| District of Columbia, | 6 | 25 |
| New Brunswick, | 1 | 1 |
| England, | 22 | 24 |
| Scotland, | 4 | 5 |
| France | 2 | 2 |
| Holland, | 1 | 1 |
| West Indies, | 1 | 1 |
| Total, | 348 | Total, 767 |

### VALUABLE REPRINT.

A translation of the excellent Treatise on First Dentition, of M. Baumes of Paris, by Professor Bond of the Baltimore College of Dental Surgery, we are happy to announce as the next work selected for republication in this Journal, to be commenced in the next number of the present volume.

The learned author was a member of many distinguished literary and scientific institutions, as will be evident from the Title page of his work which we give entire in its native language.

Traité de la Première Dentition et des maladies souvent trè-graves qui en dépendent ; ouvrage que la Société royale de Médecine de Paris couronne en 1782, et dans lequel on trouve la meilleure maniére de conduire et d'èlever les enfans de naissance. Par M. Baumes, Professeur de Pathologie et de Nosologie à l'Ecole de Medecine de Montpellier, et ci-devant Professeur de Medicine et de Clinique de l'Université de Médecine de cette ville ; ex-President et Secrétaire perpêtuel de la Société de Medecine pratique de Montpellier ; Associé de l'Ecole de Medicine de Paris ; Membre de l'Académie de Médecine, de la Société départmentale de la Société medicale d'Emulalion, de la Société Académique des Sciences et de la Société galvanique de Paris ; des Sociétés de Mèdecine de Bordeaux, de Marseilles, de Nancy, de Bruxelles, de Nismes ; des Sociétés des Sciences pe Montpellier, de Dijon, de Vaucluse, du Gard, etc. etc. A Paris, Chez Méquignon l'aîné, Libraire de l'Ecole et de la Société de Médecine, rue de Ecole de Médecine, in no 3 ou 9, vis-a-vis la rue Hautefeuille. 1806

This treatise on First Dentition, which we propose to present to our subscribers in an English version from the pen of Doctor Bond, received the medal of the Royal Medical Society, of Paris, and developes the most approved methods not only of treating the first set of teeth, but also of managing children from the period of their birth. This latter circumstance will commend this volume to the medical practitioner, and also to the general reader ; and we cannot doubt that the re-publication of such a work, together with Koecker's Dental Surgery in the remainder of the present and in the forthcoming volume, will greatly augment the list of our subscribers. But above all the dental practitioner who has the professional ambition to become acquainted with the difficulties attending the development and arrangement of the first set of teeth, and whose benevolence is sufficient to induce him to become a competent adviser of parents on this subject, will avail himself gladly of so easy an access to a vast amount of valuable instruction comprised in a work which has hitherto been a sealed book to the English reader.

There may be some members of our profession who regard the dissemination of knowledge on this and other subjects, with as jealous an eye as they do the organization of Dental Colleges, the promotion of Dental

Societies, and the establishment of dental periodicals in our country.—But there are others connected with the profession of Dental Surgery, as now practiced in this country, and in many parts of Europe, who have sufficient talent, influence and zeal to institute and perpetuate associations, magazines and colleges for the dissemination of useful knowledge, such as the wants of the profession, and the well-being of community so urgently demand :—and these men are resolved that the works of foreign authors, as well French as others, which contain important information, shall be made available to the honourable and educated practitioner, in qualifying himself to survey the entire field of dental science.

Let, then, every highminded member of our profession, come frankly and liberally to the aid of the only periodical known to exist in any of the numerous languages of the earth, for the propagation of those important truths on which our science rests. Let those who wield a ready pen write for its pages ; and let those to whom fortune has entrusted a heavy purse, subscribe for a few copies for private distribution. The bread of charity is never *uselessly* " cast upon the waters." But especially let every practicing dentist in these twenty-six States, co-operate with the publishers by forwarding at least the amount of a single subscription.—If the work be not at present all they could desire, let them improve it by their patronage. Thus shall it never again be said that a calling whose usefulness is so inseparable from the best interests of community, shall be destitute of a single periodical to advocate its cause.

The collaborators of this Publication, in addition to the Editors and Publishing Committee, are,—

H. H. Hayden, M. D. Baltimore
J. F. Flagg, M. D., Boston.
B. A. Rodriguez, M. D. Charles. S. C.
C. S. Brewster, Paris, France.
Leonard Koecker, M. D. London, Eng.
Enoch Noyes, Baltimore,
Harvey Burdell, M. D. New York.
J. P. Hullihen, M. D. Wheeling, Va.
Leonard Macall, M. D., Baltimore.
E. Maynard, M. D., Washington City.
James D. McCabe, Richmond Va.
S. M. Shepherd, Petersburg, Va.
George Merryman, Baltimore.
D. S Grandin, M. D., New-York.
W. W. H. Thackston, Va.

### AGENTS FOR THE
### AMERICAN JOURNAL OF DENTAL SCIENCE.

Doct. W. N. McKenney, *Fredericksburg, Va.*
F. B. Chewning, *Richmond, Va.*
A. Pierce, *Sussex Court House, Va.*
Chas. S. Pleasants, *Painesville, Ohio.*
David Walker & Asahel Jones, 174 *Fulton Street, New York.*
Edward Taylor *Maysville K. Y.*
O. P. Laird, M. D., *Columbus, Ga.*
John D. Chevalier, 135 *Fulton-st. N. Y.*
Geo. Washington Parmly, *N. Orleans.*
A. W. Brown, *New-York.*
F. M. Boykin, M. D. *Smithfield, Va.*
G. McDonald, M. D. *Macon, Geo.*
E. Maynard, M. D. *Washington City.*
McAllister & McNaughton, *Albany.*
M. K. Bridges, *Brooklyn.*
Blanding & Avery, *Columbia, S. C.*
Eleazer Gidney, *England.*
S. Wheeler, M. D *Murfreesboro, N. C.*
W. H. H. Davis, M. D. *Cassville, Oneida Co. New-York.*
B. A. Rodriguez, M. D. *Charles. S. C.*
Ferguson & Goddard. *Louisville, Ky.*
L. S. Stringfellow, *Baltimore.*
J. P. Norman, *Little Rock, Arkansas.*
E. E. Crowfoot, *Middletown, Conn.*
Samuel Highley, *Fleet-st. London.*

believe, only when they come very late, viz: when the jaws have left off growing. This is the want of room in the jaws for these late teeth; a circumstance which produces an addition to the other inconveniencies arising from dentition. When it takes place in the upper jaw, the tooth is often obliged to grow backwards; and in such a position it sometimes presses on the interior edge of the coronoide process, in shutting the mouth, and gives great pain. When it takes place in the lower jaw, some part of the tooth continues to lie hid under that process, and covered by the soft parts, which are always liable to be squeezed between that tooth and the corresponding tooth in the upper jaw. To open very freely, is absolutely necessary in these cases: but even that is often not sufficient. Nothing but drawing the tooth or teeth, will remove the evil in many cases.

### CASES.

It would be endless to give histories of cases, exemplifying each symptom of dentition. I shall only relate a few which are singular; and which, being extraordinary, will the better enforce the propriety, in all cases, of the cure I have recommended.

*Case* 1.—A young child was attacked with contractions of the *musculi flexores* of the fingers, and also of the toes. These contractions were so considerable as to keep her fingers and thumb constantly clinched, and so irregularly, that they appeared distorted. All the common antispasmodic medicines were given, and continued for several months, but without success.

I scarified the gums down to the teeth, and in less than half an hour all the contractions had ceased. This, however, only gave relief for a time. The gums healed; the teeth continued to grow, and filled up the new space acquired by the scarifications; and the same symptoms appeared a second time.

The former operation was immediately performed, and with the same success.

*Case* 2.—A boy, about two years of age, was taken with a pain and difficulty in making water; and voided matter from the *urethra*. I suspected that by some means or other this child might possibly be affected by the venereal poison; and the suspicion naturally fell on the nurse.

These complaints sometimes abated, and would go off altogether; and then return again. It was observed at last, that they returned only upon his cutting a new tooth: this happened so often, regularly and constantly, that there was no reason to doubt but that it was owing to that cause.

*Case* 3.—A lady about the age of five or six and twenty years, was attacked with a violent pain in the upper jaw; which at last extended through the whole side of the face, similar to a violent toothache, from a cold; and was attended with consequent fever.

It was treated at first as a cold; but, from its continuance, was afterwards supposed to be nervous.

The case was represented to me from the country; and I gave the best directions that I could, on a representation of the symptoms. She came to London some months after, still laboring under the same complaint. Upon examining the mouth, I observed one of the points of the *dens sapientiæ* ready to come through. I lanced the gums, and the disorder gave way immediately.

A lady about the same age was attacked with a violent pain in the left side of her face. It was regularly periodical; coming on at six o'clock in the evening. She took the peruvian bark, which had no effect. She took antimonials, and Dovar's powder, which also were equally ineffectual. But ene of the points of the *dens sapientæ* of the upper jaw of the same side appearing, shewed the cause, and indicated the remedy. The gums were lanced, and the pain ceased.

THE END.

---

### TO SUBSCRIBERS.

**In the next number of the American Journal of Dental Science, will be commenced the re-publication of Brown's Dentologia, a didactic Poem on the diseases of the teeth, and their proper remedies, with notes, by Eleazar Parmly. This work was originally published for private distribution, and being now out of print, it is hoped this re-publication will be acceptable to our subscribers.**

**Hunter on the Teeth, has been lately published with notes, by Thomas Bell of London, in the Select Medical Library, and Eclectic Journal of Medicine, for August 1839, vol III, No. 10; Haswell, Barrington & Haswell. Phil.**

**The following is the list of subscribers to the American Journal of Dental Science, Edited by Eleazar Parmly of New-York, and Chapin A. Harris, M. D. of Baltimore;—Publishing Committee, E. Parmly, E. Baker, S. Brown, of New-York;—of which, this re-publication is a part:—**

| | |
|---|---|
| Ayers Daniel, Amsterdam, Mont. County, N. Y. | 1 |
| Allen Rich'd L Sara. Springs, | 1 |
| Alcock James, N. Y. | 2 |
| Austin George, Baltimore, | 1 |
| Austin Nathan'l, Harrisonburg, Va. | 2 |
| Ash Claudius, London, | 2 |
| Brewster, C. S. Paris, France, | 1 |
| Burdell John, N. Y. | 20 |
| Bridges M. K. Brooklyn, | 20 |
| Baker Elisha, N. Y. | 20 |
| Brown A. W., N Y. | 20 |
| Bryan Elijah, N. Y. | 1 |
| Blake Elihu, N. Y. | 1 |
| Bancroft T. L., Granville, Ohio, | 1 |
| Brown B. B. St. Louis, Mo, | 2 |
| Backus G. Nashville, Tenn. | 1 |
| Briscoe A. W., Baltimore, | 1 |
| Barstow Wm. H., M. D., Georgetown, K. Y. | 1 |
| Buchen David L. Hampstead, Md. | 1 |
| Bidgood Richd. W., Smith. Va. | 1 |
| Badger Felix H. Decatur, Ga. | 1 |
| Brown Solyman, N. Y. | 5 |
| Becht A. J. Hague, Netherlands, | 1 |
| Bareud J. Dent., George-st., Manchester, | 1 |
| Barend Sam'l. Liverpool, Eng. | 1 |
| Bradford D. Augusta, Ky. | 1 |
| Bell Thomas, London, | 1 |
| Ballanger D. W. Montgomery, Ala. | 2 |
| Brockway Josephus, Troy, | 2 |
| Blanding S., M. D. Columbia, S. C. | 1 |
| Baldwin James Oscar, Newark, N. J. | 1 |
| Cook Doctor, Brooklyn, | 1 |
| Clute Nicholas, Louisville Ky. | 2 |
| Chandler James, Schenec. N. Y. | 1 |
| Cobb B. C., M. D. Clarkstore, Martins Co. N. C. | 1 |
| Cox A. L., M. D. New York, | 1 |

Am. J. Dental Sci.

Cleveland J. A., Charleston, S. C. 1

Crocker Fred'k, Sagharbour, L. I. 1

Clark F. H., Baltimore, 1

Comegys,—— Baltimore, 1

Cutler Wm. Daily, Baltimore, 1

Cutler Sam'l Jackson, Baltimore, 1

Coleman, R. K. Baltimore, 1

Cauling & Edmunds, Balt. 1

Cobb Anson, Brooklyn, 1

Clark C., Jacksonville, Ill. 1

Clark James, Springfield, Ill. 1

Cassill J. F., Upper Marion, Col. Mo. 1

Chewning F. B., Rich. Va. 2

Clark James, Lebanon, O. 1

Coppel Chas. Preston, Eng. 1

Crane O. P. Geneva, N. Y. 1

Chevalier J. D., N. Y. 1

Crofoot E., E. Muddletown Con. 1

Dexter, Doctor, Brooklyn, 1

Dunning H. H. Buffalo, 1

Davis W. H. H. Cassville, Oneida Co., N. Y. 1

Duncan Archibald, N. Y. 1

Davesson Frederick A., M. D. Hillsboro, Loudon Co. Va. 1

Dodge Ezra, N. Y. 1

Dunlap Joseph, Chilcothe, O. 1

Dunbar J. R. H., M. D. Balt. 1

Desha John R., M. D. Little Rock, Ark. 1

De Loude Le Chas. Wolverhampton, England, 1

Esterly D., M. D. Troy, N. Y. 1

Early ——, Doctor, Lynchburg, Va. 2

Elmendorf Joseph, Pennyan, N. Y. 1

Ellery E. Baltimore, 1

Evans J. N., M. D. Cynthiana, Ky. 1

Elliott Joseph D. Leicester, Mass. 1

Ensor — Dentist, 4 Seal-st. Liverpool, 1

Fenn Horatio N., M. D. Rochester, 2

Foster J. H., M. D. New-York, 2

Flagg Josiah F., M. D. Boston, 1

Frazer, Doctor, Cynthiana, Harrison Co. Ky. 1

Frink J. N. Portland, 1

Foote George, Vernon, N. Y. 1

Faulkner & Pierpont, Man. England, 1

Fundenberg G. B. Dentist Pittsburg, Pa. 1

Gilfiland, Doctor, Brooklyn, 1

Gaines Rich. W. Charlotte C. H. Va. 1

Garrison, Doctor, Brooklyn, 1

Gallop L. F. Newport, R. I. 1

Gardette E. B., Philadel. 2

Greenwood Isaac J., New-York, 1

Green L. T., Nashville, Tenn. 1

Goddard W. H., Louisville, Ky. 1

Gill Bryson, Baltimore, 1

Gunnell, M. D. Washington, 1

Griffith S. & E. Louisville, Ky. 1

Geer—Rev. John A., Maryland, 1

Grandhomme M. P. Paris, France, 1

Gidney Eleazar, Man. England 1

Herd, Doctor, Brooklyn, 1

Harris Chapin A., M. D. Balt. 40

Hawes Arnold, Pawtucket, R. I. 1

Hulihen J. P. Wheeling, Va. 1

Hawes & Allen, New-York, 1

Houston P., N. Y. and Charleston, 1

Hartness Thos. L. New-York, 1

Hewlet J. W., Greensbo, N. C. 1

Holmes Oliver, Baltimore, 1

Howard F., Washington City, 1

Harris John, M. D. Georgetown, Ky. 2

Hall A. S., M.D. Scotland Neck, N.C. 1

Hubbard, E. R., Newbern, N. C. 1

Harper Sam'l, Kent Island, Md. 1

Hard G. C., Easton, Pa. 1

Humphreys Geo. W., Winchester, Va. 1

Hodgson, Doctor, Whiteplains, N. Y. 1

Harrison R.H , M.D. Huntsville, Ala. 1

Imrie—Dentist. Man. England, 1

Johnson Wm., Hagerstown, Md. 2

Jenks W. D., Fredrickstown, Md. 2

King, Doctor. Brooklyn, 1

Kelley Elbridge G., Newburyport, Mass. 2

Knower Daniel, N. Y. 1

Keene Benjamin F., Hills. Ga. 1

Knapp, F. H. Baltimore, 1

Latham Hiram, Brooklyn, 1

Lum John Patterson, N. J. 1

Levett M., New York, 1

Lovejoy John, New-York, 1

Lossing B. J. New York, 1

Laroque Edward, Baltimore, 1

Lapham B. B., Baltimore, 1

Lawrence J., M. D., Tarboro, N. C. 1

Leadbetter John, Alexan. D. C. 1

Lyon S. K., N. Orleans, 4

Lloyd T. B., Manchester, Eng. 1

Lloyd Rich., Liv. Eng. 1

Laird O. P., Columbus, Ga. 1

Lamphire Wm., Alexan. D. C. 1

Marvin, Doctor, Brooklyn 1

Miller Seth P., Worcester, Mass. 1

Maynard E. —. ~~M. D.~~ Washington City 20

Martin Chas. F., Norfolk, Va. 1

M'Kinney W.N., Fredericksburg, Va. 20

Milhau John, New York, 1

Munson W. G. New Haven, 1

Manly Horace, Cananda. N. Y. 1

Macall Leonard, M. D., Balt. 1

Miller J. H. Professor, &c. Baltimore 1

Merryman Geo. Baltimore, 1

Manning M. E., Tarborough, N. C. 2

M'Cabe James D., Richmond, Va. 2
McDonald G., M. D. Macon, Ga. 2
Macey Wm. M., M. D. Georgetown, Ky. 1
McAllister J. M. Albany, 1
McNaughton M. A. Albany, 1
Mason, Doctor, Brooklyn, 1
Merritt C., Bridgport, Con. 1
Nelson Alexander, Albany, 1
Norman S. P., Little Rock, Ark. 4
Overfield M. Winchester, Va. 2
Parker, Doctor, Brooklyn, 1
Parmly L. S., N. Orleans, 20
Parmly Jahial, New-York, 20
Parmly Jahial Savannah, 1
Parmly Geo. W., N. Orleans. 1
Parmly David, New York, 1
Parmly Eleazar, New York 40
Parmly Ludolph, Mobile, 1
Parmly W. Samuel, New York, 1
Patello Wm. H., Charlotte C.H. Va. 1
Park David N., A. M. New-York, 1
Parkhurst Wm. H., New York, 1
Peak J. M. Cooperstown, N. Y. 1
Plant Eleazar, Willimantic, Conn. 1
Pritchard P. C., Jackson, N. C. 1
Plough A. L., New Orleans, 1
Parsons J, H., Liverpool, England, 1
Palmer W. A., New Haven, Conn. 1
Pleasants Charles, Painesville, O. 1
Royce W. A. Poughkeepsie, N. Y. 3
Robinson E. C. Norfolk, Va. 1
Roper Lewis, M. D. Philad. 20
Rowell Chas., N. Y. 2
Roe Early, M. D. Hillsborough, Ga. 1
Rodriguez B. A., Charles. S. C. 1
Ritter — Washington City, 1
Robinson E. M. D. Leesburg, Ky. 1
Reynolds Wm., M. D. Camden, S. C.
Strickland Benj. Cleveland, O. 1
Snow R. J., Buffalo, 1
Smith & Thackston, Farmville, Va. 1
Scott W. R., Raleigh, N. C. 4
Shuff P. L., M. D., Leesburg, Ky. 1
Smeds, M. D., Frankfort, Ky. 1
Stringfellow S. L. Baltimore, 20
Stratton Cha's, Keene N. H. 1
Sanders M., London, 1
Sanborn E., Andover, Mass. 1
Smith Wm., Liverpool, Eng. 1
Stinkley B. D., Albany, 1
Smith G. W., N. Orleans, 1
Stuart R., Knoxville, E. T. 1
Smith J. W. Amherst, Mass. 1
Searles F., Springfield, Mass. 1
Stockton S. W., Phila. 20
Taylor Edward, M.D., Bainbridge, O. 2
Tucker E. G., M. D. N. Y. 2
Trenor John, M. D. N. Y. 1
Trenor James, M. D., N. Y. 1
Tilyard H. W., Baltimore. 1
Taliaferro T.S., M. D., Maysville, Ky. 1
Thompson — Doctor, Colum. O. 1
Thorn, Doctor, Brookyln, 1
Tyler Nah'l S. Mass. Chicapee Falls, 1
Taylor James, Crawfordville, Ia. 1
Teter G. T., Dentist, Greenfield, Highland Co. Ohio, 1
Van Praag A. S., N. Y. 1
Vincent Ezra, N. Y. 1
Van Camp, Louisville, Ky. 1
Van Patten C. H. 1
Vanboskirk L. E., St. John, N. B. 1
Willard M. T. Concord, N. H. 1
Weed J. Sacket, W. Greenfield, N. Y. 1
White Geo. H., N. Y. 1
Walker & Jones, N. Y. 20
Wanzer N. C., Auburn, N. Y. 1
Wayt John G., Ricmond Va. 2
Wilson J. D. Richmond, Va. 2
Ware W., M. D. Wilmington, N. C. 1
Wheat J. B., N. Haven, 1
Young H., Troy, N. Y. 1

If each of our subscribers would obtain an additional name to be inserted in this catalogue, the permanency of this Journal would be amply secured. As it is, the Publishing Committee assure the profession, that the first volume of twelve numbers, shall be fully completed without any unnecessary delay. The long interim between the present and the last number, has been occasioned by the difficulty of transmitting available funds from remote parts of the country.

Our special patrons are again requested to forward either the whole, or a part of their subscription, to Mr. Jahial Parmly, Bond-street, New-York, Treasurer—and our advertising friends are invited to remember the Printer.

[In nos. 9-10.]

### LIST OF SUBSCRIBERS.

The republication of the foregoing Poem and Notes, as a part of several successive numbers of the American Journal of Dental Science, for the years 1840–41, was sustained by the following catalogue of subscribers:—

| | |
|---|---|
| Ayers Daniel, Amsterdam, Mont. County, N. Y. | 1 |
| Allen Richard L., Sara. Springs, | 1 |
| Alcock James, N. Y. | 2 |
| Austin George, Baltimore, | 1 |
| Austin Nathan'l, Harrisonburg, Va. | 2 |
| Ash Claudius, London, | 2 |
| Azling Isaac, M D., Boston, | 1 |
| Auten F. P., Lambertville, N. J. | 1 |
| Atkinson John, Leeds, Eng. | 1 |
| Arthur Robert, Baltimore, | 1 |
| Andrews E. H., Charlotte C. H., N. Carolina, | 1 |
| Ash G. E., London, | 1 |
| Arnold Wm., M. D., N. York, | 1 |
| Avery Samuel, N. Orleans, | 1 |
| Brewster, C. S. Paris, France, | 1 |
| Burdell John, N. Y. | 20 |
| Bridges M. K. Brooklyn, | 20 |
| Baker Elisha, N. Y. | 20 |
| Brown A. W., N. Y. | 20 |
| Bryan Elijah, N. Y. | 1 |
| Blake Elihu, N. Y. | 1 |
| Bancroft T. L., Granville, Ohio, | 1 |
| Brown B. B. St. Louis, Mo. | 2 |
| Backus G. Nashville, Tenn. | 1 |
| Briscoe A. W., Baltimore, | 1 |
| Barstow Wm. H., M. D., George-town, Ky. | 1 |
| Buchan David L. Hempstead, Md. | 1 |
| Bidgood Richd. W. Smith, Va. | 1 |
| Badger Felix H. Decatur, Ga. | 1 |
| Brown Solyman, N. Y. | 5 |
| Becht A. J. Hague, Netherlands, | 1 |
| Bareud J. Dent., George-st., Manchester, | 1 |
| Barend Sam'l. Liverpool, Eng. | 1 |
| Bradford D. Augusta, Ky. | 1 |
| Bell Thomas, London, | 1 |
| Ballanger D. W. Montgomery, Ala. | 2 |
| Brockway Josephus, Troy, | 2 |
| Blanding S., M. D., Columbia, S. C. | 1 |
| Baldwin James Oscar, Newark, N. J. | 1 |
| Brown Chas. D., Philadelphia, | 1 |
| Burdell Harvey, M. D., *N.* York, | 2 |
| Bliss S., M. D., Syracuse, N. York, | 1 |
| Buck J. B., *N.* York, | 1 |
| Briscoe Jas H, Philadelphia, | 1 |
| Boyken F M, Smithfield Va | 1 |
| Burr Hudson, Philadelphia, | 1 |
| Ballard Geo. W, Madison, Morgan Co, Georgia, | 1 |
| Bull Abel, M D, Boston, | 1 |
| Bemis S A, Boston, | 1 |
| Budd John D, Mount Holly, N J, | 1 |
| Burr Wm H, Mount Holly, N J, | 1 |
| Cook Doctor, Brooklyn, | 1 |
| Clute Nicholas, Louisville, Ky. | 2 |
| Chandler James, Schenec. N. Y. | 1 |
| Cobb B. C., M. D., Clarkstore, Martins Co., N. C. | 1 |
| Cox A. L., M. D., New York, | 1 |
| Cleveland, J. A., Charleston, S. C. | 1 |
| Crocker Fred'k, Sagharbour, L. I. | 1 |
| Clark, F. H., Baltimore, | 1 |
| Comegys, —— Baltimore, | 1 |
| Cutler Wm. Daily, Baltimore, | 1 |
| Cutler Sam'l Jackson, Baltimore, | 1 |
| Coleman, R. K. Baltimore, | 1 |
| Cauling & Edmunds, Baltimore, | 1 |
| Cobb Anson, Brooklyn, | 1 |
| Clark C., Jacksonville, Ill. | 1 |
| Clark James, Springfield, Ill. | 1 |
| Cassill J. F., Upper Marion, Col. Mo. | 1 |
| Chewning F. B., Rich. Va. | 2 |
| Clark James, Lebanon, O. | 1 |
| Coppel Chas. Preston, Eng. | 1 |
| Crane O. P. Geneva, N. Y. | 1 |
| Chevalier, J. D. N. Y. | 1 |
| Crofoot E., E. Middletown Con. | 1 |
| Crofoot L L, Middletown, Conn, | 1 |
| Candee J G, Troy, N York, | 1 |
| Clark A, Penyan, N Y, | 1 |
| Cameron James, Philadelphia, | 1 |
| Copeland W S, M D, Rich Square, Virginia, | 1 |
| Carter J H, Ravena, Portage Co, O, | 1 |
| Caldwell Geo H, Rushville, Ia, | 1 |
| Camp W C, Oxford, Granville Co, N Carolina, | 1 |

Cuyler Vernon, M D, Hartford, Conn, 1

Caldwell D, Philadelphia, 1

Copelin J, New-York, 1

Crane W S, Hartford, Conn, 1

Culp Clark, Philadelphia, 1

Dexter, Doctor, Brooklyn, 1

Dunning H. H. Buffalo, 1

Davis W. H. H, Cassville, Oneida Co., New York. 1

Duncan Archibald, N. Y. 1

Davesson Frederick A., M. D. Hillsboro, Loudon Co. Va. 1

Dunlap Joseph, Chilicothe, O. 1

Dunbar J. R. H., M. D., Balt. 1

Desha John R., M. D., Little Rock, Arkansas, 1

De Loude Le Chas. Wolverhampton, England, 1

Dodge J Smith, New-York, 1

Dewar Henry, Edinburgh, Scot, 2

Dodge Andrew, Matanzas, Cuba, 1

Dixon Rufus E, M D, Boston, 1

Doolittle A B, Plymouth, Conn, 1

Esterly D., M. D., Troy, N. Y. 1

Early ——, Doctor, Lynchburg, Va. 2

Elmendorf Joseph, Pennyan, N. Y. 1

Ellery E. Baltimore, 1

Evans J. N., M. D., Cynthiana, Ky. 1

Elliott Joseph D., Leicester, Mass. 1

Ensor—Dentist., Liverpool, 1

Ellis Calvin, M D, Boston, 1

Evans G W, Cincinnatti, 1

Epps W J, M D, Langhorne's P O, Virginia, 1

Evans Thos W, Philadelphia, 1

Easton Wm T, Providence, R I, 1

Fenn Horatio N., M. D., Rochester, 2

Foster J. H., M. D., New-York, 2

Flagg Josiah F M., M. D., Boston, 1

Frazer Doctor, Cynthiana, Harrison Co., Ky. 1

Follen John H, North. C. H., Va, 1

Frink J. N., Portland, N. H. 1

Foote George, Vernon, N. Y. 1

Faulkner & Pierpont, Man. England, 1

Fundenberg G. B., Dentist, Pittsburgh, Pa. 1

Franklin B W, Fairfield, Herkimer Co, New-York, 1

Fraetas J A, New-York, 20

Fay Timothy, Baton Rouge, La, 1

Fay Solomon, Chester Factories, Mass, 1

Ferguson Jas H, Northumberland C House, Va. 1

Fouche W. W. Philadelphia, 1

Falconer John, New-York, 1

Gilfiland, Doctor, Brooklyn, 1

Gaines Rich. W., Charlotte C. H., Virginia, 1

Garrison, Doctor, Brooklyn, 1

Gallop L. F., Newport, R. I. 1

Gardette E. B., Philadelphia, 2

Green L. T., Nashville, Tenn. 1

Goddard W. H., Louisville, Ky. 1

Gill Bryson, Baltimore, 1

Gunnell Jas. S., M. D., Washington, 1

Griffith S. & E., Louisville, Ky. 1

Geer—Rev. John A., Maryland, 1

Grandhomme M. P., Paris, France, 1

Gidney Eleazar, Man., England, 1

Greenwood Isaac J, New-York, 40

Grant C W, Newburg, New-York, 1

Greenleaf Chas, Hartford, Conn, 1

Ganson Holton, Batavia, New-York, 1

Gaines B B, Cassville, Georgia, 1

Githens John H, Philadelphia, 1

Gunn ——, Nashville, Tennessee, 1

Grimes Doctor, Greenborough, Ga. 1

Herd, Doctor, Brooklyn, 1

Harris Chapin A., M. D., Balt. 40

Hawes, Arnold, Pawtucket, R. I. 1

Hulihen J. P., Wheeling, Va. 1

Hawes & Allen, New-York, 1

Houston P., N. Y. and Charleston, 1

Hartness Thos. L., New-York, 1

Hewlet J. W., Greensbo, N. C. 1

Holmes Oliver, Baltimore, 1

Howard F., Washington City, 1

Harris John, M. D. Georgetown, Ky. 2

Hall, A. S., M. D., Scotland Neck, N. Carolina, 1

Hubberd, E. R., Newbern, N. C. 1

Harper Sam'l, Kent Island, Md. 1

Hand G. C., Easton, Pa. 1

Humphreys Geo. W. Winchester, Virginia, 1

Hodgson Doctor, Whiteplains, N. Y. 1

Harrison R. H., M. D., Huntsville, Alabama, 1

Helsby ——, Manchester, England, 1

Hudson Edward, Philadelphia, 1

Hamlin T B, Wythvelle, Virginia, 1

Hallified Doctor, Petersburg, Va, 1

Hought Chas. J, Philadelphia, 1

Hughes H W, Westminster, Md, 1

Hill A, Norwalk, Connecticut, 1

Hoit Emmett M, Stanwich, Conn, 1

Halleyman W F, Maytinton, S C, 1

Imrie—Dentist, Man. England, 1

Ingraham Thomas, Philadelphia, 1

Johnson Wm., Hagerston, Md. 2

Jenks W. D., Frederickstown, Md. 2

J'ollen John N., York Co. H. Va. 1

King Doctor, Brooklyn, 1
Kelley Elbridge G., Newburyport, Mass. 2
Knower Daniel, N. Y. 1
Keen Benjamin F., Hills, Ga. 1
Knapp, F. H. Baltimore, 1
Kearsing George, New-York, 20
Kingsbury C A, Philadelphia, 1
Kimball Horace, New-York, 1
Keemy B M, Hudson New-York, 1
Koecker Leonard, M D, London, 2
Keene, Doct. Newtown, Scott Co, Kentucky, 1
Latham Hiram, Brooklyn, 1
Lum John, Patterson, N. J. 1
Levett M., New York, 1
Lovejoy John, New York 1
Laroque Edward, Baltimore, 1
Lapham B. B., Baltimore, 1
Lawrence J., M. D., Tarboro, N. C. 1
Leadbetter John, Alexan. D. C. 1
Lyon S. K., N. Orleans, 4
Lloyd T. B., Manchester, Eng. 1
Lloyd Rich., Liv. Eng. 1
Laird O. P., Columbus, Ga. 1
Lamphire Wm., Alexan. D. C. 1
Lawrence S W, Philadelphia, 1
Lee Joseph, M D, Camden, S C, 1
Lawrence E B, Crawfordville, Ga, 1
Loomis J C, Carlisle, Pa, 1
Latimer James, Madison, Morgan Co, Georgia, 1
Lethbridge Sam'l, Richmond, Va, 1
Marvin, Doctor Brooklyn, 1
Miller Seth P.,, Worcester, Mass. 1
Maynard E. — M. D. Washington City 20
Martin Chas. F., Norfork, Va. 1
M'Kinney W.N,,Fredricksburg, Va. 20
Milhau John, New York, 1
Munson W. G. New Haven, 1
Manly Horace, Canada. N. Y. 1
Macall Leonard, M. D. Balt. 1
Miller J. H. Professor, &c. Baltimore 1
Merryman Geo. Baltimore, 1
Manning M. E., Tarborough, N. C. 2
M'Cabe James D., Richmond, Va. 2
McDonald G., M. D. Macon, Ga. 2
Macey Wm. M., M. D. White Sulphur Scott C. Ky. 1
McAllister J. M. Albany, 1
McNaughton M. A. Albany, 1
Mason, Doctor, Brooklyn, 1
Merritt C., Bridgeport, Con. 1
Martin J, Portsmouth, England, 1
Mac Pherson James, Glasgow, Scot. 1
Middleton Ellis, Philadelphia, 1
Mc Ilhinney Joseph E, Philadelphia, 1
Matson Alpheus, Auburn, New-York, 1
MGrath R, Philadelphia, 1
More Justus E, Philadelphia, 1
Middleton Wm. New-York, 1
Merritt Charles, Bridgeport, Conn, 1
Murrill L, Petersburg, Va, 1
Nelson Alexander, Albany, 1
Norman S. P., Little Rock Ark. 4
Neal D, Philadelphia, 1
Noyes Enoch, Baltimore, 20
Nasmyth Robert, Edinburgh, Scot. 1
Overfield M. Winchester, Va. 2
Parker, Doctor, Brooklyn, 1
Parmly L. S., N. Orleans, 20
Parmly Jahial, New-York, 20
Parmly Jahial, Savannah, 1
Parmly Geo. W., N. Orleans, 1
Parmly David, New York, 1
Parmly Eleazar, New York, 40
Parmly Ludolph, Mobile, 1
Parmly W. Samuel, New York, 1
Patello Wm. H.,Charlottee C. H. Va. 1
Park David N., A. M. New York, 1
Parkhurst Wm. H., New York, 1
Peak J. M. Cooperstown, N. Y. 1
Plant Ebenezer, Willimantic, Conn. 1
Pritchard P. C. Jackson, N. C. 1
Plough A. L., New Orleans, 1
Parsons J. H., Liverpool, England, 1
Palmer W. A., Stratford, Conn. 1
Pleasants Charles, Painesville, O. 1
Perkins Jacob, Springfield, Mass, 1
Parker T H, Philadelphia, 1
Pent George, M D, Lawrenceville, Virginia, 1
Pancost S, Shelbyville, Kentucky, 1
Royce W. A. Poughkeepsie, N. Y. 3
Robinson E. C. Norfolk, Va. 1
Roper Lewis, M. D. Philad. 20
Rowell Chas., N. Y. 2
Roe Early, M. D. Hillsborough, Ga. 1
Rodriguez B. A. M. D. Charles, S. C. 1
Ritter—Washington City, 1
Robinson E. M. D. Leesburg, Ky. 1
Reynolds Wm., M. D. Camden, S. C. 1
Reinstein Frederick, Philadelphia, 1
Ross Samuel, New-York, 1
Root J B, Hamilton, Madison Co, New-York, 1
Reese F. A. M. D. Hamilton Bermuda. 1
Strickland Benj. Cleveland, O. 1
Snow R. J., Buffalo, 1
Smith & Thackston, Farmville, Va. 1
Scott W. R., Raleigh, N. C. 4
Shuff P. L., M. D., Leesburg, Ky. 1
Sneeds M. D., Frankford Ky. 1

Stringfellow S. L. Baltimore, 20

Stratton Cha's, Keene, N. H. 1

Sanders M., London, 1

Sanborn E., Andover, Mass. 1

Smith Wm., Liverpool, Eng. 1

Stinkley B. D. Albany, 1

Smith G. W., N. Orleans, 1

Stuart R., Knoxville, E. T. 1

Smith J. W. Amherst, Mass. 1

Searle F., Springfield, Mass. 1

Stockton S. W. Philadelphia, 20

Stowell John, Philadelphia, 1

Shepherd S *M*, Petersburg, Va, 1

Sherman A, Newark, New-Jersey, 2

Simpson ——, *M*anchester. England, 1

Sale T A, Williamsboro, N C, 1

Sims J *M*, M D, Fish Dam, Union *D*is, S Carolina, 1

Sanders E, 16 Argyle st, London, 1

Stevens B H, Elbridge, Onondagua Co, New-York, 1

Taylor, Edward, M. D. Bainbridge, O. 2

Tucker, E. G., M. D., New-York, 2

Trenor John, M. D., New York. 1

Trenor *J*ames, *M. D., N. Y.* 1

*T*ilyard *H. W. B*altimore 1

*T*aliaferro *T. S., M. D. M*ays. *K*y. 1

*T*hompson — *D*octor, *C*olum. *O.* 1

*T*horn, *D*octor, *B*rooklyn 1

*T*yler *N*athaniel, *M*ass. *C*hicapee *F*alls, 1

*T*aylor *J*ames, *C*rawfordville, *I*a. 1

*T*eter *G. T., D*entist, *G*reenfield, *H*ighland *C*o. *O*hio. 1

Thorn Doctor, Edgefield C H, S C, 1

Thackston Doctor, Farmville, Va, 1

Townsend Sam'l, Baltimore, 1

Truman, George, Philadelphia, 1

*V*an *P*raag *A. S., N*ew *Y*ork, 1

*V*incent *E*zra, *N*ew *Y*ork, 1

*V*an *C*amp, *L*ouisville, *K*y. 1

*V*an *P*aten *C. H., P*ittsburg, 1

*V*anboskirk *L. E. S*t *J*ohn, *N. B.* 1

*W*illard *M. T. C*oncord, *N. H.* 1

*W*eed *J. S*acket, *W.*Greenfield, *N.Y* 1

*W*hite *G*eo. *H., N*ew *Y*ork, 1

*W*alker & *J*ones, *N*ew *Y*ork, 20

*W*anzer *N. C., A*uburn, New *Y*ork, 1

*W*ayt *J*ohn *G- R*ichmond, *V*a, 2

*W*ilson *J. D., R*ichmond, *V*a, 2

*W*are *W, M. D., W*ilmington, *N.C.* 1

*W*heat *J. B., N*ew *H*aven, 1

White, S D, Philadelphia, 1

Worthington R C, M D, Murrfreesboro, N C, 1

Ward David G, Wanesboro, N C, 1

Ward W A, Petersburg, Va, 2

Willmore E, Baltimore, 1

Wheeler E D, Hillsboro, Coffee Co, Tennessee, 1

Williams E C, M D, *P*hiladelphia, 1

Wells H, Hartford, Conn, 1

*Y*oung *H., T*roy, *N*ew *Y*ork, 1

Yard George, *P*hiladelphia, 1

